# Comment on ‘Lung cancer in the Swiss HIV Cohort Study: role of smoking, immunodeficiency and pulmonary infection’

**DOI:** 10.1038/bjc.2012.181

**Published:** 2012-05-03

**Authors:** A Bearz, E Vaccher, R Talamini, M Berretta, U Tirelli

**Affiliations:** 1 Department of Medical Oncology, National Cancer Institute of Aviano (PN), Via Franco Gallini 2, Aviano (PN) 33081, Italy; 2 Epidemiology and Biostatistics Unit, National Cancer Institute of Aviano (PN), Aviano (PN), Italy

**Sir,**

We read with great interest the paper ‘Lung cancer in the Swiss HIV Cohort Study: role of smoking, immunodeficiency and pulmonary infection’ by Clifford *et al* (2012) about the characteristics of HIV patients with a diagnosis of lung cancer. In particular, they found out how in their cohort lung cancer was not associated with CD4+ cell count nadir, history of AIDS and use of c-ART as well. On behalf of the Italian Cooperative Group on AIDS and Tumors (GICAT) we performed a 25-year survey, between November 1986 and March 2009, collecting 70 consecutive patients with lung cancer and HIV infection, of whom 34 (50%) belonged to the era before the introduction of c-ART into clinical practice and the remaining 36 (52%) to the era after it became available. In Table 1 we summarise the clinical characteristics of the patients.

Our patients were mostly intravenous drug users (overall, 57%) and males (90%); the overall median age was 43.5 years. All patients were heavy smokers (⩾20 packs per year), except for one who did not smoke at all and three patients for whom data were not available; overall 94% of the patients were smokers.

Median CD4+ count nadir did not differ significantly in both groups, with 278 cells *μ*l^−1^ (12–987) in the pre-c-ART cohort and 339 cells *μ*l^−1^ (4–761) in the post-c-ART group: overall, 19 patients (27%) showed a CD4+ nadir <200 cells *μ*l^−1^, 11 (22%) >500 *μ*l^−1^ and 30 (42%) within 200–499 *μ*l^−1^. Data about the viral load at the time when lung cancer was diagnosed are available only for the post-c-ART group of patients; in most cases, 21 out of 36 (58%), it was undetectable, while in 5 patients (14%) it was ⩾10 000 copies per ml, in 2 patients (6%) 500–9999 copies per ml, and in 8 patients (22%) <500 copies per ml.

These data confirm that lung cancer in HIV is not associated with immunodeficiency; as already pointed out by other investigators (Powles *et al*, 2003), this might suggest that the immune function has less of a role in the pathogenesis of lung cancer than in Kaposi’s sarcoma or non-Hodgkin’s lymphomas.

The overall survival rate was significantly better for the post-c-ART group, 3.8 months *vs* 7 months, *P*=0.01, in the pre- and post-c-ART group, respectively. However, the cause of death was comparable between both groups, with lung tumour being the leading cause. The two groups differed for performance status (PS) at the time of presentation, with poorer PS score in the pre-c-ART patients. As the PS at the time of diagnosis is one of the strongest predictors of overall survival for lung cancer, it is likely that the better outcome for the post-c-ART group may depend on better PS at diagnosis of lung cancer. When the cause of death was lung cancer, overall survival was significantly better for the post-c-ART patients (7 *vs* 4.1 months in the post- and pre-c-ART group, respectively); for example, the cancer-related mortality rate at 1 year was 85% for the pre-c-ART patients *vs* 67% for the post-c-ART patients.

With our data we strongly confirm the association between smoking and lung cancer in HIV patients, the lack of association of immunodeficiency, as well as an improvement of survival in the post-c-ART period, likely from a gain in PS score.

## Figures and Tables

**Table 1:** Clinical characteristics of 70 HIV-infected patients with lung cancer by time of treatment (pre-c-ART ⩽1996 and post-c-ART >1996)

**Treatment**	**Pre-c-ART (** ***n*** **=34)**	**Post-c-ART (** ***n*** **=36)**	***P***
*Age (years)*			
<45	23 (67.4)	18 (50.0)	ns
⩾45	11 (32.3)	18 (50.0)	
			
*Sex*			
M	31 (91.2)	32 (88.8)	ns
F	3 (8.8)	4 (11.8)	
			
*Risk*			
IVDU	23 (67.6)	17 (47.2)	ns
Homosexual	6 (17.6)	10 (27.7)	
Other	5 (14.7)	9 (25.0)	
			
*Smoke*			
Yes	32 (94.3)	34 (94.5)	ns
No	2 (5.7)	2 (5.5)	
			
*Histology*			
SCC	8 (23.5)	9 (25.0)	
ADK	13 (38.2)	19 (52.3)	
LCC	5 (14.7)	6 (16.6)	
Small cell	8 (23.5)	2 (5.5)	
			
*PS*			
0–1	19 (55.9)	25 (69.4)	0.02
⩾2	15 (44.1)	11 (30.5)	⩾2

Abbreviations: ADK=adeno-carcinoma; c-ART=combined antiretroviral therapy; IVDU=intravenous drug users; LCC=large-cell carcinoma; ns=non-significant; PS=performance status; SCC=squamous cell carcinoma.
